# Comorbidity, limitations in activities and pain in patients with osteoarthritis of the hip or knee

**DOI:** 10.1186/1471-2474-9-95

**Published:** 2008-06-26

**Authors:** Gabriella M van Dijk, Cindy Veenhof, Francois Schellevis, Harry Hulsmans, Jan PJ Bakker, Henk Arwert, Jos HM Dekker, Guus J Lankhorst, Joost Dekker

**Affiliations:** 1 NIVEL (Netherlands Institute for Health Services Research), Utrecht, The Netherlands; 2 HAGA Ziekenhuis, Department of Rheumatology, The Hague, The Netherlands; 3 Jan van Breemen Institute, Amsterdam, The Netherlands; 4 Medisch Centrum Alkmaar, Department of Rehabilitation, Alkmaar, The Netherlands; 5 Sophia Revalidatie centrum, The Hague, The Netherlands; 6 Department of Rehabilitation Medicine, EMGO Institute, VU University Medical Centre, Amsterdam, The Netherlands; 7 Department of General Practice, EMGO Institute, VU University Medical Centre, Amsterdam, The Netherlands

## Abstract

**Background:**

This study aims to contribute to the knowledge of the influence of comorbidity in OA. The objectives of the study were (i) to describe the prevalence of comorbidity and (ii) to describe the relationship between comorbidity (morbidity count, severity and the presence of specific diseases) and limitations in activities and pain in elderly patients with knee or hip OA using a comprehensive inventory of comorbidity.

**Methods:**

A cross-sectional cohort study was conducted, in which 288 elderly patients with hip or knee osteoarthritis were included. Apart from demographic and clinical data, information about comorbidity, limitations in activities (WOMAC, SF-36 and timed walking test) and pain (VAS) was collected by questionnaires and tests. Statistical analyses included descriptive statistics, multivariate regression techniques, *t*-tests and one-way ANOVA.

**Results:**

Almost all patients suffered from at least one comorbid disease, with cardiac diseases, diseases of eye, ear, nose, throat and larynx, other urogenital diseases and endocrine/metabolic diseases being most prevalent. Morbidity count and severity index were associated with more limitations in activities and with more pain. The presence of most of the moderate or severe diseases and obesity was associated with limitations in activities or with pain.

**Conclusion:**

The results of this study emphasize the importance of comorbidity in the rehabilitation of elderly patients with osteoarthritis of the hip or knee. Clinical practitioners should be aware of the relationship of comorbidity with functional problems in OA patients.

## Background

Elderly patients with osteoarthritis (OA) frequently suffer comorbidity. Comorbidity refers to the coexistence of other conditions with a defined index disease [1,2]. OA is one of the diseases with the highest rate of comorbidity [1,3-6]. Patients with OA have a significantly higher risk of developing comorbidity than non-OA patients [4,7]. Studies focussing on comorbidity in OA showed that chronic conditions, such as hypertension, cardiovascular diseases, obesity, respiratory diseases and diabetes can be found alongside OA [1,3-5,8].

There is evidence that comorbidity is related to disability in general populations [9-13]. Studies on the relationship between comorbidity and disability in OA showed similar results [3,5,8,14]. Morbidity count (the number of diseases) was associated with limitations in activities in end stage hip osteoarthritis [5]. In a population recruited from general practice, an association between morbidity count and pain or quality of life in patients with hand, knee or hip OA was established [3,8]. Likewise, an effect of comorbidity on quality of life and limitations in activities was found in patients that were placed on a waiting list for total hip replacement [14]. Also, longitudinal studies provided evidence for a relationship between comorbidity and limitations in activities [15,16]. Obesity, an important health hazard, can be regarded as a comorbidity and has been found to be associated with limitations in activities, body functions and quality of life in OA [11,17,18].

Apart from the studies on obesity, none of the former studies assessed the relationship between the presence of specific diseases and limitations in activities. This is surprising since some diseases, in combination with OA, might be associated with limitations in activities, whereas others might not [19]. The majority of the studies on comorbidity focussed on morbidity count. Comorbidity lists in these studies varied with regard to the number of diseases included in the list and the type of diseases that were studied. Studies often only focussed on the most prevalent diseases. Furthermore, in none of the studies severity of the comorbid conditions was taken into account. A comorbidity measure that includes all possible disease categories and severity is the Cumulative Illness Rating Scale (CIRS) [20]. Thus, the CIRS allows a more in depth analysis of comorbidity in OA than is available in previous studies.

The objectives of the study were (i) to describe the prevalence of comorbidity and (ii) to describe the relationship between comorbidity (morbidity count, severity and the presence of specific diseases) and limitations in activities and pain in elderly patients with knee or hip OA using a comprehensive inventory of comorbidity.

## Methods

### Design

A cross-sectional study was conducted in 288 patients with knee or hip OA. The study was approved by the Medical Ethical Committee of the VU University Medical Centre, Amsterdam, the Netherlands.

### Study population

Patients were recruited from three rehabilitation centres and two hospitals (departments of Orthopaedics, Rheumatology or Rehabilitation). The present study is part of a larger research program on rehabilitation of elderly patients with OA of the hip or knee. For this reason, we focussed on rehabilitation centres and hospitals in recruiting patients. Inclusion criteria were: (a) diagnosis of hip or knee OA by medical specialist according to radiological criteria (only if X-rays were available) or clinical criteria of the American College of Rheumatology; (b) 50 years of age or older; (c) referral to hospital or rehabilitation centre less than a year before inclusion; (d) at least moderate functional problems (Lequesne algofunctional index score ≥ 5) [21] and (e) informed consent. Exclusion criteria were: (a) 85 years of age or older; (b) insufficient understanding of the Dutch language and (c) expected death within one year after inclusion due to fatal illness.

Initially, 775 patients with osteoarthritis of the hip or knee that visited the department in the year prior to inclusion were contacted by mail and were asked to participate in the study. Of those patients that volunteered (n = 364), 288 were included. 76 patients were excluded because they did not meet the inclusion criteria. Reasons of exclusion were age (n = 2), language (n = 4), less than moderate functional problems (n = 48) and referral longer than one year before inclusion (n = 22). Analyses showed that there were no differences between the group of patients that were initially contacted (N = 775) and the patients that were included in the study (N = 288) with regard to age and gender. Some differences were found in the location of OA. Compared to our study population, patients that were initially contacted suffered less frequently from both hip and knee OA (6.2%) and more frequently from knee OA (59.5%) and hip OA (34.3%).

### Measurements

Measurements were carried out by means of tests, questionnaires and interviews. Assessments were performed on test locations by the researcher or the research assistant.

#### Demographic and clinical data

Demographic and clinical data were collected for each patient including age, gender, height, weight, location of OA, duration of complaints, other joint complaints, level of education and marital state. If available, X-rays of the hip and knee that were recorded in the year prior to inclusion were scored on joint space width and osteophytes, following a standardized procedure [22,23]. A 0–3 scale was used for rating the radiographs: 0 = normal; 1 = mild or 1–33% abnormal; 2 = moderate or 34–66% abnormal; 3 = severe or 67–100% abnormal. Data on radiological impairments were summarized into one score for the hip and one score for the knee. This summary score was determined by the highest score on either joint space width or osteophytes.

#### Limitations in activities and pain

Limitations in activities were measured using the physical functioning subscale of the MOS 36 item Short Form Health Survey (SF-36) [24,25], a 10 meter timed walking test [26] and the physical functioning subscale of the Western Ontario and McMaster Universities Osteoarthritis Index (WOMAC) [27,28]. Pain was rated at the time of assessment on a Visual Analogue Scale (VAS) (range 0–10). A higher score on SF-36 and the WOMAC reflects fewer limitations in activities, whereas a higher score on the VAS means more pain.

#### Comorbidity

**Figure 1:**
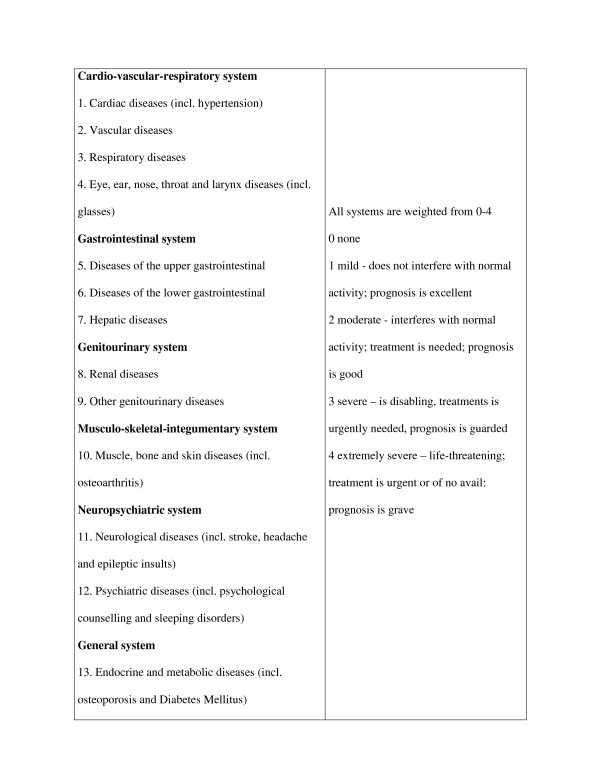
The Cumulative Illness Rating Scale (CIRS).

Information about comorbidity was gathered in an interview with the patient using the Cumulative Illness Rating Scale (CIRS) [20,29,30]. The CIRS consists of 13 domains related to different body systems. Examples of diseases included in the different categories of the CIRS were given in the description of the instrument. Scoring on the different domains is weighted by the severity of the comorbid condition. Severity scores range from 0 (none) to 4 (extremely severe). More details about the CIRS are presented in Figure 1. Because all patients suffered from OA and this disease is used as the index disease, CIRS category #10 (muscle, bone and skin diseases) was excluded from the analyses. Indices of comorbidity that were derived from the CIRS were the presence (0/1) of the different disease categories, morbidity count and the severity index (sum score on the CIRS divided by morbidity count). With regard to morbidity count two calculations were made: (i) the number of diseases on which the patients scored 1 or higher and (ii) the number of diseases on which the patients scored 2 or higher (moderate or more severe comorbidity). Finally, obesity was added to the list of comorbidities and was defined by a Body Mass Index (body weight divided by length^2^), BMI ≥ 30 kg/m^2^.

### Statistical analyses

Descriptive statistics were used to describe the presence and severity of comorbidity and limitations in activities and pain. Furthermore, the relation between comorbidity and limitations in activities and pain was determined using multivariate linear regression analyses. Dependent variables were the WOMAC physical functioning score, SF-36 physical functioning score, the timed walking test and the VAS pain score. Comorbidity variables (morbidity count and severity index) and possible confounders or effect-modifiers (age and sex) were entered stepwise. Only those variables that influenced both comorbidity and functioning were added into the regression analyses as confounders. Correlations showed that apart from age and gender, other variables such as BMI, marital state, education and other musculoskeletal disorders, were not associated with comorbidity and functioning.

If significant interaction was identified (p < 0.05), stratified analyses were performed. *T*-tests for independent samples were performed to analyse differences in limitations and pain between patients that suffered from specific diseases (all separate disease categories in the CIRS) and those who did not suffer from these diseases. The presence of a specific disease was defined by a score of 2 or more on the CIRS. Oneway-ANOVA was used to analyse differences in limitations in activities and pain between patients with normal weight, overweight patients and patients with obesity. The 11.5 version of SPSS was used.

## Results

### Study population

Baseline characteristics of the study population are presented in Table 1. The majority of the patients (80%) originated from departments of Orthopaedics. The other 20% came from departments of Rheumatology and departments of Rehabilitation.

**Table 1:** Baseline characteristics

Gender: male, n (%)	83 (28.8%)
N = 288	
Age, mean (sd)	66 (8.7)
N = 287	
Location of OA, n (%)	
N = 287	
• knee	139 (48.4%)
• hip	72 (25.1%)
• both	76 (26.5%)
Duration of complaints (years), mean (sd)	10.1 (10.7)
N = 283	
Radiological impairments in the hip *	
• Normal	2 (2.2%)
• Mild	4 (4.4%)
• Moderate	13 (14.5%)
• Severe	71 (78.9%)
Radiological impairments in the knee **	
• Normal	6 (4.3%)
• Mild	8 (5.8%)
• Moderate	7 (5.1%)
• Severe	117 (84.8%)

* N = 90 (from only a part of the included patients X-rays were available); ** N = 138 (from only a part of the included patients X-rays were available)N, n: number; sd: standard deviation; OA: osteoarthritis.

### Limitations in activities and pain

The mean score for physical functioning was 45.15 (sd = 21.62) on the SF-36 physical functioning domain (range 0–100) and 61.01 (sd = 17.76) on the WOMAC physical functioning domain (range 0–100). The mean number of seconds for the 10 meter timed walking test was 10.36 (sd = 4.11). The mean pain score was 4.81 (sd = 2.56) on the VAS.

### Comorbidity

For detailed results on comorbidity, the reader is referred to Table 2 and 3. Almost all patients (98.6%) suffered from one or more coexistent diseases and 84.4% of the study population suffered from one or more moderate or severe coexistent diseases (CIRS ≥ 2). The most prevalent conditions in this OA population were cardiac diseases (54%), eye, ear, nose, throat and larynx diseases (96.1%), urogenital diseases (44.4%) and endocrine and metabolic diseases (46%).

**Table 2:** Comorbidity according to CIRS *

	Mild or more severe comorbidity **	Moderate or more severe comorbidity ***
Morbidity count mean (sd) N = 288	4.32 (2.06)	2.60 (1.93)
0–12		

Number of diseases n (%)		
○ None	4 (1.4%)	45 (15.6%)
○ 1 or 2	55 (19.1%)	98 (34.1%)
○ 3–6	144 (50%)	121 (42%)
○ 6–9	79 (27.4%)	22 (7.6%)
○ ≥9	6 (2.1%)	2 (0.7%)

* CIRS 10 (Muscle, bone and skin diseases) was excluded from the CIRS; ** number of diseases on which patients scored ≥ 1; *** number of diseases on which patients scored ≥ 2/none of the patients reported a score of 4 on the CIRS;

CIRS: cumulative Illness Rating Scale; sd: standard deviation; N, n: number of patients.

**Table 3:** The presence of comorbidity, measured by disease categories of the CIRS and their severity

	Presence (patients that reported to have comorbidity (score 1, 2 or 3)) n (%)	Severity median* (25^th ^percentile; 75^th ^percentile), range
Cardiac diseases	154 (54%)	2.00 (2.00; 2.00), 0–3
Vascular diseases	73 (25.6%)	2.00 (1.00; 2.00), 0–3
Respiratory diseases	82 (28.8%)	2.00 (1.00; 2.00), 0–3
Ear, eye, throat and larynx diseases	274 (96.1%)	1.00 (1.00; 2.00), 0–3
Gastro-intestinal diseases (upper part)	99 (34.7%)	2.00 (1.00; 2.00), 0–2
Gastro-intestinal diseases (lower part)	87 (30.5%)	2.00 (1.00; 2.00), 0–3
Liver diseases	21 (7.4%)	1.00 (1.00; 2.00), 0–3
Renal diseases	32 (11.2%)	1.00 (1.00;2.00), 0–2
Urogenital diseases	126 (44.4%)	1.00 (1.00; 2.00), 0–3
Diseases of bones, joints, muscle and skin	285 (100%)	2.00 (2.00; 2.00),1–3
Neurological diseases	91 (31.9%)	2.00 (1.00; 2.00), 0–3
Psychiatric diseases	75 (26.3%)	2.00 (1.00; 2.00), 0–3
Endocrine and metabolic diseases	131 (46%)	2.00 (2.00;2.00), 0–3

N = 285; *Calculated for all patients that suffered from a specific disease. CIRS: Cumulative Ilness Rating Scale: N, n: number of patients.

Furthermore, the mean BMI was 27.8 (sd = 4.5). The majority (51.7%) of the patients were overweight (BMI between 25 and 30) and 23.6% suffered from obesity. Only 13% of the patients had no other joint complaints. Frequently occurring other joint complaints were hand and back problems, respectively 56.3% and 65.6%. Analyses for the knee and hip OA separately revealed similar results.

### Association between comorbidity and limitations in activities and pain

As Table 4 shows, morbidity count was associated with limitations in activities on the WOMAC and on the SF-36. In older patients (age 72–84), a significant relationship was established between morbidity count and worse performance on the timed walking test. Morbidity count was also associated with more pain measured on the VAS.

**Table 4:** Results from the multivariate regression analyses: association between comorbidity (morbidity count (CIRS ≥ 1), morbidity count (CIRS ≥ 2), severity index) and limitations in activity (WOMAC, SF-36, timed walking test) and pain (VAS) ^Δ^*

	Morbidity count	Morbidity count	Severity index
	(CIRS ≥ 1)	(CIRS ≥ 2)	
WOMAC (physical functioning domain)	-0.208‡	-0.323‡	-0.322‡
	R^2 ^= 0.056	R^2 ^= 0.114	R^2 ^= 0.114

SF-36 (physical functioning domain)	-0.286‡	-0.350‡	-0.281‡
	R^2 ^= 0.114	R^2 ^= 0.152	R^2 ^= 0.113

Timed walking test			
Age 50–60 **			0.148†
Age 61–71	0.169	0.184∫	
Age 72–84	0.123	0.179∫	
	0.262†	0.338‡	
	R^2 ^= 0.139	R^2 ^= 0.185	R^2 ^= 0.097

VAS (pain)	0.167‡	0.233‡	0.230 ‡
	R^2 ^= 0.032	R^2 ^= 0.058	R^2 ^= 0.056

^Δ ^Standardized β's are presented *All multivariate associations are corrected for age and gender. Other corrections were found to be unnecessary. ** A significant interaction was found between morbidity count and age (age categories were chosen in order to have equal distribution of patients over the categories). ∫ 0.1 < p < 0.05; † p < 0.05; ‡ p < 0.01. WOMAC: Western Ontario and MacMaster universities osteoarthritis index; SF-36: MOS 36 item Short Form Health Survey; VAS: Visual Analogue Scale.

Table 4 also shows a significant relationship between severity index and self reported limitations in activities on the WOMAC and the SF-36. Severity index was also significantly associated with less functional performance on the timed walking test. Furthermore, severity index was associated with more pain on the VAS.

Although significant associations were found, comorbidity only accounted for a small part of the variance in limitations in activities and pain. R^2^ranged from 0.032 to 0.139 for morbidity count, from 0.058 to 0.185 for morbidity count of moderate or severe diseases and from 0.056 to 0.114 for severity index.

In summary, morbidity count (having more diseases and having more moderate or severe diseases) and severity index were associated with more limitations in activities and with more pain. Comorbidity, however, accounts for only a small percentage of the variance in limitations in activities and pain. Analyses for knee and hip OA separately reveal similar results.

### Association between the presence of specific diseases and limitations and pain

Because a stronger association was found for morbidity count with CIRS score ≥ 2 than for morbidity count with CIRS score ≥ 1 and because moderate or severe comorbidity accounted for a larger part of the variance, Table 5 shows the mean differences in scores for limitations in activities and pain between patients that suffer from moderate or severe coexistent diseases (CIRS score ≥ 2) and patients that do not. Most of the moderate or severe diseases and obesity were found to be associated with limitations in activities (WOMAC, SF-36 and timed walking test) or with pain (VAS).

**Table 5:** Limitations in activities and pain (WOMAC, SF 36, timed walking test, VAS) in patients who suffer moderate or severe coexistent diseases (CIRS* and obesity**) and patients who do not.

	WOMAC (PF)	SF 36 (PF)	Timed walking test	VAS (pain)
Cardiac disease				
yes	31.14	40.84‡	10.46	5.14
no	28.42	48.66	10.27	4.57
Vascular disease				
yes	33.66†	36.44‡	11.05	5.61†
no	28.77	46.98	10.21	4.66
Respiratory disease				
yes	31.82	39.46†	10.44	5.14
no	29.18	46.32	10.34	4.76
Eye, ear, nose throat and larynx disease				
yes	32.75‡	40.82†	10.88	4.99
no	28.11	47.20	10.10	4.75
Disease gastro-intestinal system (upper part)				
yes	34.36‡	37.05‡	10.65	5.15
no	28.13	47.66	10.26	4.73
Disease gastro-intestinal system (lower part)				
yes	35.25‡	35.26‡	12.64†	5.41
no	28.48	47.13	9.91	4.71
Hepatic disease				
yes	41.46†	29.26	17.16	7.50‡
no	29.40	45.43	10.21	4.77
Renal disease				
yes	32.23	47.22	9.88	5.23
no	29.53	44.99	10.38	4.81
Other genito urinary disease				
yes	34.61‡	37.26‡	12.19‡	5.20
no	28.23	47.35	9.84	4.73
Neurological disease				
yes	32.33	41.17	11.49	5.37†
no	28.84	46.30	10.02	4.66
Psychiatric disease				
yes	34.20‡	34.80‡	11.43	5.26
no	28.58	47.55	10.11	4.73
Endocrine/metabolic disease				
yes	31.94†	41.71†	11.16†	5.09
no	28.32	47.03	9.89	4.68
Body weight				
Normal weight	25.38	20.41	9.84	4.43
Overweight^Δ^	30.78	18.65	10.25	4.87
Obesity^ΔΔ^	31.56†	18.50†	10.74	5.04

*t-test;** one way ANOVA; † p < 0.05; ‡ P < 0.01; ^Δ^(25 ≤ BMI < 30); ^ΔΔ^(BMI ≥ 30) BMI: Body Mass Index; WOMAC: Western Ontario and MacMaster universities osteoarthritis index; SF-36: MOS 36 item Short Form Health Survey; VAS; Visual Analogue Scale; PF: physical functioning; CIRS: Cumulative Illness Rating Scale.

## Discussion

The objectives of the study were (i) to describe the prevalence of comorbidity and (ii) to describe the relationship between comorbidity and imitations in activities and pain in elderly patients with knee or hip OA using a comprehensive inventory of comorbidity. In this study we focussed on the cross-sectional relationship between the presence of coexistent diseases according to the CIRS and limitations inn activities and pain, measured by recommended instruments [31], in patients with hip or knee OA. Also, the presence of obesity was taken into account.

A study in the Netherlands showed that in a general population of people aged 55 years and older, the mean score on the SF-36 ranged from 60.0 (age > 85) to 72.7 (age 55–65) [32]. So, compared to this population, the patients in this study reported more limitations in activities (a score of 45.15 on the subscale physical functioning of the SF-36).

Almost all patients (98.6%) suffered from at least one comorbid disease, with cardiac diseases, diseases of ear, eye, throat and larynx, urogenital diseases and endocrine/metabolic diseases being most prevalent. The prevalence of comorbidity in this study population is high, compared to earlier research. A likely explanation is that diseases which do not interfere with normal life and which have an excellent prognosis (CIRS score 1) also contribute to the prevalence. For example wearing glasses or contact lenses is seen as comorbidity since these patients score 1 on CIRS #4. If the prevalence of comorbidity is calculated for diseases that interfere with normal activity and with a poorer prognosis (CIRS ≥ 2), then the prevalence is 84.4%. This is still higher than reported in earlier studies on comorbidity in OA. A potential explanation is that in this study a comprehensive list for comorbidity, which includes all possible disease categories, is used. In earlier research, limited lists of comorbidity were applied [3,15,33]. Marks, for example, used a list of five diseases: hypertension, cardiovascular diseases, peripheral vascular diseases, diabetes and respiratory diseases [5]. It can be noted that for example the presence of diseases of ear, eye, throat and larynx diseases and urogenital diseases, which are most prevalent in the present study, were not studied by Marks. There were, however, studies that applied longer lists [8]. But these list were restricted, in the sense that they did not use broad categories, but simply focussed on specific diseases, for example, gallstones, prostate hypertrophy and migraine, not taking into account all other possible diseases within a category (such as other urogenital diseases (e.g. incontinence) and other neurological diseases (e.g. stroke)).

In the present study, evidence is provided that comorbidity is negatively associated with limitations in activities and pain. These results confirm earlier research, in which morbidity count was associated with disability, pain and quality of life [3,5,8]. Furthermore, this study showed that the presence of most of the coexistent moderate or severe diseases was related to limitations in activities and pain. Like in this study, obesity has been found to be associated with disability in earlier cross-sectional studies in OA [11,17] and has been identified as a risk factor for disability in longitudinal prognostic research [34]. Furthermore it has been reported that obesity is an important factor in the origin of radiological changes in osteoarthritis, either by mechanical or metabolic processes [35]. Taking this into account, the reduction of obesity and weight loss might be an important treatment modality in OA. In a recent study, a combination of weight loss and exercise therapy has proven to be successful in improving self-reported functioning. However, this study reported no positive effect on radiological changes [36]. In the present study, also psychiatric disease was associated with limitations in activities. This finding strengthens evidence from earlier research in OA in which psychological factors, such as anxiety and depression, were reported to be associated with pain and [37]. Furthermore, good mental health is considered as a protective factor for functional decline [34].

Some methodological issues need to be considered. Firstly, because all patients suffered from OA and the influence of comorbidity in an OA population was studied, CIRS category #10 was excluded from the analyses. CIRS category #10 does not only include OA, but also other musculoskeletal and skin diseases. Consequently, information about those types of diseases from the CIRS cannot be presented or included in the analyses. In an attempt to give some information about other joint complaints a question, which comprised of neck, shoulder, hand, back, foot and other complaints, was added. Analyses revealed that patients who suffer other joint complaints, in particular neck complaints, report more limitations in activities on the SF-36 and the WOMAC. They also reported more pain on the WOMAC and the VAS. Secondly, in this study self-reported comorbidity, rather than comorbidity assessed by a physician, was used. One should be aware, that self-reported comorbidity could be influenced by personal or mood characteristics. However, positive correlations were found between self-reported comorbidity measures and comorbidity from medical record reviews [38]. Thirdly, of the 775 patients who were initially contacted, 364 patients volunteered to participate and 288 patients were included in the study. Non-response analyses showed that there were no differences in age and gender between the patients who were contacted and the patients who were finally included in our study. Some differences were found in location of OA. But since, results of the study did not differ between patients with hip OA and patients with knee OA, non-response was not expected to influence the results. Fourthly, one can debate whether all diseases have the same impact on functioning and result in the same burden. The comparability of equal scores on CIRS might provide a problem in assessing the influence of comorbidity. Fifthly, results were only corrected for age and gender and not for other variables such as BMI, marital status, education and other musculoskeletal disorders, since correlations showed that these variables were not correlated with comorbidity and functioning. These variables, therefore, were not considered as confounders. Lastly, the influence of the disease on daily activities is part of the definition of severity (see figure 1). Some of the variance in the relationship between severity and functional problems can be attributed to the definition of severity in the CIRS. Scoring severity on the CIRS is determined by both interference with normal life and medical issues, such as prognosis and treatment. Separating those two aspects in scoring severity of comorbidity seems advisable. In all, in spite of its metric flaws, until now no better, comprehensive instrument on comorbidity is available.

## Conclusion

Patients included in this study were recruited from hospitals and rehabilitation centers. Results are important for this specific group of patients, since it is expected that with the aging of the population an increasing number of elderly people with osteoarthritis will need rehabilitation.

The prevalence of comorbidity in this population of elderly patients with hip or knee OA was high and an association between comorbidity and limitations and pain was established. Comorbidity, however, accounted for only a small percentage of the variance and can therefore not be considered as the only important aspect in an elderly population that may contribute to limitations in activities and pain. Other aspects related to aging, such as physical impairments (radiological changes, muscle strength and range of motion) and cognitive impairments, but also social network and psychosocial variables, are expected to play an important role in limitations in activities and pain in elderly patients with hip or knee OA. More scientific knowledge of the impact of those factors is needed and further research should elaborate on these aspects.

The results of this study emphasize the importance of comorbidity in the rehabilitation of elderly patients with osteoarthritis of the hip or knee. Clinical practitioners should be aware of the relationship of comorbidity with functional problems in OA patients.

## Competing interests

The authors declare that they have no competing interests.

## Authors' contributions

GMvD contributed to conception and design of the study, the acquisition of data, the analyses and interpretation of data and drafting and revising the manuscript; CV contributed to the analyses and interpretation of data, drafting and revising the manuscript and gave final approval of the manuscript; FS contributed to the analyses and interpretation of data, drafting and revising the manuscript and gave final approval of the manuscript; HH contributed to the acquisition of data and revising the manuscript and gave final approval of the manuscript; JPJB contributed to the acquisition of data and revising the manuscript and gave final approval of the manuscript; HA contributed to the acquisition of data and revising the manuscript and gave final approval of the manuscript; JHMD contributed to the acquisition of data and revising the manuscript and gave final approval of the manuscript; GJL contributed to conception and design of the study and revising the manuscript and gave final approval of the manuscript; JD contributed to conception and design of the study, analyses and interpretation of data and drafting and revising the manuscript and gave final approval of the manuscript.

## Pre-publication history

The pre-publication history for this paper can be accessed here:


